# Association between Oral Conditions and Returning Home after Discharge in Elderly Patients

**DOI:** 10.3390/geriatrics2030028

**Published:** 2017-08-25

**Authors:** Koji Naruishi

**Affiliations:** Department of Periodontology and Endodontology, Institute of Biomedical Sciences, Tokushima University Graduate School, 3-18-15 Kuramoto, Tokushima 770-8504, Japan; naruishi@tokushima-u.ac.jp; Tel.: +81-88-631-3111; Fax: +81-88-633-7009

**Keywords:** elderly, oral frailty, cognitive impairment, destination after discharge

## Abstract

For elderly inpatients, it may be preferable to return back to their homes after discharge. Therefore, it is important to predict whether elderly patients are able to return home after discharge. Our purpose is to examine the association between geriatric factors including oral conditions and returning home after discharge in elderly patients. A total of 257 elderly patients were enrolled (returned home: *N* = 116; changed to hospital/nursing home: *N* = 141). Oral conditions were evaluated by trained dentists. Cognitive impairment was evaluated using the clinical dementia rating scale. Odds ratios (OR) and 95% confidence intervals (CI) to predict the destination after discharge were obtained by unconditional logistic regression analysis. Impaired tongue movement and edentulous were significant oral factors that elderly patients cannot return home (Impaired tongue movement, OR: 2.72; Edentulous, OR, 1.89), whereas presence of loss of posterior occlusion and mobile teeth were not associated statistically. Cognitive impairment, but not aftereffect of cerebrovascular disease, was a significant problem to predict the destination after discharge in elderly patients (Cognitive impairment, OR: 3.58; Cerebrovascular disease, OR: 1.27). Simple, reliable and inexpensive evaluation including oral examination may better predict whether elderly patients can return home after discharge.

## 1. Introduction

Japan is a steadily aging society, and the number of hospitalized elderly patients has been increasing [1]. In many elderly patients, they are very pleased to return back to their home after hospital discharge. However, various geriatric factors, i.e., lower body mass index (BMI), diminished physical recovery, oral frailty, aftereffect of cerebrovascular disease and cognitive impairment are closely related to each other in elderly patients, resulting in development of unwanted lethal diseases such as aspiration pneumonia [2,3]. Although it is well known that the maintenance of good oral hygiene is important to reduce the risk of aspiration pneumonia, the oral hygiene of elderly patients is often very poor because of disabled limbs by several geriatric factors such as aftereffect of cerebrovascular disease or cognitive impairment [3].

Although severity of the diseases must be considered as a significant factor to decide the destination after hospital discharge, it may be preferable for elderly patients to return back to their homes after hospital discharge. Socially, it is also important that elderly patients return home after discharge, because an increase of elderly patients in hospital may weaken the country’s economic strength. Therefore, in view of the residence of elderly patients after discharge, it is very important to predict whether elderly inpatients can return to their home after discharge, because their family can prepare a good care plan in advance.

The destination of the elderly after hospital discharge is generally decided by a medical doctor’s judgement. Although the decision might depend on the disease severity, it is very significant for their family to predict easily whether the elderly patients can return to their home after hospital discharge. In the present study, we examined the association between geriatric factors including oral conditions and returning home after discharge in elderly patients. We believe our approach would contribute to the planning of good care for elderly people in caregivers, including their families, in the future.

## 2. Methods

### 2.1. Subjects and Evaluation

A total of 257 elderly patients admitted to Tottori Municipal Hospital between January 2013 and December 2013 were enrolled (returned home: *N* = 116 [M/F:51/65], average age: 84.0 ± 6.0 yrs; changed to hospital/nursing home: *N* = 141 [M/F:48/93], average age: 85.6 ± 6.5 yrs). Although a total of 262 elderly patients were enrolled at first, 257 elderly patients were analyzed, because 5 patients passed away while in hospital. Physical ability of the patients was assessed using Functional Independence Measure (FIM) scores at admission. Oral conditions of elderly patients were evaluated by inspection and palpation including stability of the posterior occlusion, tongue movement, number of teeth and presence of mobile teeth. For the determination of posterior occlusion stability, it was defined in patients with normal posterior teeth or dentures as “stable”. All of the oral examinations were performed by trained dentists. Cognitive impairment of elderly patients was evaluated using the clinical dementia rating scale (CDR) [4]. All examinations were performed when the condition of elderly patients became stable after acute medical care. This study was approved by the ethics committee of Tottori Municipal Hospital (No. 1153). All procedures performed in the present study involving human participants were in accordance with the ethical standards of the institutional and/or national research committee and with the 1964 Helsinki Declaration.

### 2.2. Statistical Analyses

Statistical analyses were performed using JMP^®^ 8 version 8.0.2 (SAS Institute Japan, Tokyo, Japan). Statistical differences of age, length of hospitalization and FIM score were analyzed using the Mann–Whitney U test. The odds ratios (OR) derived from a bivariate analysis and 95% confidence intervals (CI) of the several factors due to which the elderly patients cannot return to their home after discharge were obtained by unconditional logistic regression analysis. In all statistical analyses, *p*-values < 0.05 were considered statistically significant.

## 3. Results

No statistical difference was observed between groups of returned home and changed hospital/nursing home after discharge in their average age (*p* = 0.054, Mann–Whitney U test), although the average age of the group of changed hospital/nursing home after discharge was relatively higher (Table 1). Length of hospitalization in the group of elderly who changed hospital/nursing home was statistically longer than in the elderly who returned home (*p* = 0.0007, Mann–Whitney U test). FIM score in the group of elderly who changed hospital/nursing home was statistically lower than in the elderly who returned home (*p* < 0.0001, Mann–Whitney U test).

As shown in Table 2, impaired tongue movement and edentulous were significant oral factors to predict that the elderly patients cannot return home after hospital discharge (Impaired tongue movement: OR, 2.72, 95% CI, 1.5–5.0, *p* = 0.0011; Edentulous: OR, 1.89, 95% CI, 1.1–3.2, *p* = 0.017), whereas presence of loss of posterior occlusion and mobile teeth were not associated with the destination after discharge (Loss of posterior occlusion: OR, 1.67, 95% CI, 0.9–3.0, *p* = 0.093; Mobile teeth: OR, 1.11, 95% CI, 0.56–2.2, 0.76). These results would propose the clinical usefulness of simple, visual oral examination and collaboration with a dentist to predict the residence of elderly patients after discharge. Furthermore, cognitive impairment and aspiration pneumonia were significant systemic geriatric factors to predict that the elderly patients cannot return home after hospital discharge (OR, 2.47, 95% CI: 1.3–4.6, *p* = 0.0034), whereas presence of aftereffect of cerebrovascular disease was not associated with the destination (OR, 1.27, 95% CI: 0.77–2.1, *p* = 0.35). Importantly, cognitive impairment was the most significant factor due to which the elderly patients cannot return home after discharge (OR, 3.58, 95% CI: 2.1–6.0, *p* < 0.0001).

## 4. Discussion

In geriatric medicine, an increase in medical expenses for elderly patients in hospital must be a serious problem worldwide [1]. The FIM motor scale is a test used clinically to assess their rehabilitation outcome and activity of daily living (ADL) [5]. Recently, we calculated statistically FIM scores to determine the cut-off values as to whether elderly patients were able to return home after discharge [6]. Our results showed that the FIM scores of elderly females who returned home after discharge were higher than those of elderly males. Although the difference in sex may be an important factor to predict whether elderly patients can return home, in the present study, no significant difference was observed between males and females in the destination after discharge (*p* = 0.10, chi-square test). Furthermore, as expected, FIM score in the group of elderly who changed hospital/nursing home was statistically lower than in the elderly who returned home. To ensure association of destination decided by the difference in sex, further statistical analysis will be needed because of complicated causes by various factors such as physical or economic problems, although ADL of the elderly must be a significant factor.

As shown in Table 2, we have demonstrated for the first time that impaired tongue movement and edentulous, which relates to oral muscle strength, were significant predictors that the elderly patients cannot return home after discharge. It has been well known that chronic periodontitis is a polymicrobial infectious disease [7] and the disease may result in loss of teeth by inflammation-mediated bone resorption [8]. Therefore, edentulous is an unexpected consequence of the periodontitis, resulting in their impaired masticatory muscle. Since it is relatively easy to evaluate whether or not the elderly patients are edentulous, edentulous might be a useful parameter to predict the destination after hospital discharge, even in their families. Exactly, although we found that loss of posterior occlusion and presence of mobile teeth were not significant factors, we propose the importance of periodontitis treatment to prevent teeth loss in middle-aged people, because the loss of posterior occlusion and mobile teeth are clinical findings seen in the halfway point until edentulous [9]. Next, we found that cognitive impairment, but not aftereffect of cerebrovascular disease, is a significant problem that predicts that elderly patients cannot return home. We have considered that impaired cognition might be a more serious problem than aftereffect of cerebrovascular disease, because of sometimes unpredictable speech and action. This present situation must be a very significant problem socially, because elderly patients with impaired cognition have been increasing steadily. A series of findings support the clinical usefulness of oral examination and collaboration with a dentist to predict the destination after discharge in elderly patients. Of course, since it remains unknown whether the improvement of oral frailty by denture wearing or oral muscle training can lead the elderly patients to their home, further investigation will be needed to prove the clinical significance of oral function.

This study has several limitations. First, because all patients were assessed at our hospital, the subjects of this study are inherently limited and the results may not reflect the actual situation of elderly people in every country. A multicentre, prospective study with a larger number of elderly patients would be necessary to confirm these results. Second, although we posit a bidirectional relationship, we are only able to prove associations and not causation. Third, no adjustments were made for other factors which may affect the disease severity such as cancer chemotherapy, medication, smoking, etc. We believe that a risk adjustment approach could increase the validity of this study.

Taken together, the geriatric factors including oral frailty were significant problems to predict the unwanted destination after hospital discharge in Japanese elderly people. For the judgement of the destination after hospital discharge, the condition of being orally frail, such as impaired tongue movement, might be a useful factor, and the viewpoint would contribute to the proposal of a care plan to their families for quality end-of-life care for the elderly. Our findings may be useful information for both elderly inpatients, including their family, and medical experts toward their return home after hospital discharge. These findings also indicate the clinical usefulness of oral examination and collaboration with a dentist to predict the destination after hospital discharge of elderly people in the end-of-life stage.

## Figures and Tables

**Table 1:** Patients characteristics.

	Returned Home	Changed Hospital/Nursing Home	*p*-Value
*N* (M/F)	116 (51/65)	141 (48/93)	
Age (yrs)	84.0 ± 6.0	85.6 ± 6.6	0.054
Length of hospitalization (days)	30.8 ± 22.6	36.8 ± 27.0	0.007 *
FIM score	56.1 ± 23.6	34.6 ± 16.0	<0.0001 *

*N* = 257. Although a total of 262 elderly patients were enrolled at first, 257 elderly patients were analyzed, because 5 patients passed away while in hospital. FIM, Functional Independence Measure. *p*-value, Mann–Whitney U test. *, *p* < 0.05 was considered statistically significant.

**Table 2:** Several factors due to which the elderly patients cannot return home after discharge.

Factors	Odds Ratio	95% CI	*p*-Value
Oral factors			
Loss of posterior occlusion	1.67	0.9–3.0	0.093
Impaired tongue movement	2.72	1.5–5.0	0.0011 *
Edentulous	1.89	1.1–3.2	0.017 *
Presence of mobile teeth	1.11	0.56–2.2	0.76
Systemic geriatric factors			
Aftereffect of cerebrovascular disease	1.27	0.77–2.1	0.35
Aspiration pneumonia	2.47	1.3–4.6	0.0034 *
Cognitive impairment	3.58	2.1–6.0	<0.0001 *

*N* = 257. The odds ratios (OR) derived from a bivariate analysis and 95% confidence intervals (CI) of the several factors due to which the elderly patients cannot return to their home after discharge were obtained by unconditional logistic regression analysis. *p*-value, chi-square test. *, *p* < 0.05 was considered statistically significant. CI, confidence intervals.
